# Supplementary material for: Knowledge and practices of exclusive breastfeeding among mothers in rural areas of Rajshahi district in Bangladesh: A community clinic based study
Source: PLoS One. 2020 May 8;15(5):e0232027. doi: 10.1371/journal.pone.0232027 (PMC7209213; doi:10.1371/journal.pone.0232027)
Supplement: Attachment — Submitted filename: Response to Reviewers.docx [file pone.0232027.s002.docx]

**Point wise response to academic editor comments:**

1. I ensure that our manuscript meets PLOS ONE‘s style.
2. We uploaded questionnaire of
i) Original/Mother’s language.
ii) English language as supporting information.
3. The authors declare that they have no competing interest.

DASCOH Foundation is nonprofit organization and they have no any fund for publication and its supporting document is provided

4. Figure 1 is omitted from the manuscript
5. SPSS data file is uploaded in Data Availability statement
6. We have already ensured the affiliation of each author
7. It is already deleted
8. Table 3 is inserted in the respective section in the text of the manuscript (in the line of 206 in Manuscript file)

**# 1. Reviewer comments:**

1. BMC Pediatric. 2018; 18:93 is already studied. Some variable are same and some are different but the outcome variable is converted into composite index, then it is classified into two groups as good and poor.

“Dependent variable is converted to composite index”- This study worked on EBF in Rajshahi district as well as community clinic based study. Most of the researchers studied on EBF practices but this study worked about knowledge and practices on EBF. One of the novelty of this study was to introduce the composite index on EBF. “To the best of our knowledge none study has been conducted on EBF” this section is deleted from the manuscript

2. Actually the data of this study is primary collected during the period of September to December 2015. Consequently this data are not extracted from Bangladesh Demographic and Health Survey (BDHS) 2014. In fact, this study is not part of BDHS 2014.
3. According to World Bank national accounts data, and OECD National Accounts data files’2015 per capital income in Bangladesh 1,248.453 US dollar its exchange rate for December 31, 2015, 1 US dollar = 78.9031 BDT. For this reason we divided monthly family income into two categories such as ≤ 9,999 BDT and ≥ 10,000 BDT.
4. Adjusted odds ratio (AOR) and Bangladeshi taka (BDT) is corrected in the abstract as well as in the respective sections (in the line 43 and 49 of Manuscript).
5. We carefully check and revise the respective portions of discussion or results.
6. Since relevant information are giving in the respective table, so we did not provide figures.
7. We have compared our finding to the previous mentioned article name International Breastfeeding Journal 9,7(2.014) in the Introduction section of Manuscript
8. We have carefully checked the whole manuscript thoroughly.

**# 2. Reviewer Comments:**

1. The studies of Bangladesh as well as South East Asia on EBF are added in the line of 79-81.

2. Methodology section was revised thoroughly for proper explanation and understanding.

3. The article was rewriting for making article crispy and easy to understand that were indicated in the Results section

4. The discussion section was edited minutely, carefully and comprehensively.

5. Conclusion is elaborately rewriting based on the findings in terms of knowledge and practices of EBF.
